# Nilotinib and imatinib: potential candidates for treatment of dementia and Parkinson’s disease through national health insurance data

**DOI:** 10.3389/fneur.2025.1628876

**Published:** 2025-08-26

**Authors:** Jihun Song, Sun Jae Park, Yu-Jin Kim, Sang Min Park

**Affiliations:** ^1^Department of Biomedical Informatics, Korea University College of Medicine, Seoul, Republic of Korea; ^2^Biomedical Research Center, Korea University Guro Hospital, Seoul, Republic of Korea; ^3^Department of Biomedical Sciences, Seoul National University Graduate School, Seoul, Republic of Korea; ^4^Center for Biomaterials, Biomedical Research Institute, Korea Institute of Science and Technology, Seoul, Republic of Korea; ^5^Department of Family Medicine, Seoul National University Hospital, Seoul, Republic of Korea

**Keywords:** Parkinson’s disease, tyrosine kinase inhibitors, drug repositioning, dementia, nilotinib, imatinib (gleevec)

## Abstract

**Background:**

Real-world evidence on the potential of tyrosine kinase inhibitors (TKIs) for dementia and Parkinson’s Disease (PD) is crucial. This observational study aimed to evaluate TKIs, particularly nilotinib and imatinib, as potential therapeutic agents for these conditions.

**Methods:**

In this retrospective cohort study, 5,579 cancer patients who were prescribed TKIs (users; ≥ 40 years) within 5 years were used, while propensity score-matched patients without any record of TKIs (never users) served as the reference. An association of TKIs with dementia and PD was assessed by the Fine-Gray Model with adjusted-competitive hazard ratios (aCHRs) and 95% confidence intervals (CIs): [aCHRs (95% CIs; *p*-value)].

**Results:**

The risk of dementia decreased when all types of TKIs [0.65 (0.48–0.88; <0.01)], imatinib [0.66 (0.48–0.89; <0.01)], and nilotinib [0.46 (0.23–0.93; <0.05)] was used in cancer patients. Additionally, the reduced risk of PD was identified in users of all [0.56 (0.33–0.97; <0.05)] and imatinib [0.55 (0.32–0.96; <0.05)]. When the risk was evaluated according to the number of times for total usage, the aCHRs for PD in the low, middle, and high-frequency groups were 0.46 (0.20–1.02), 0.78 (0.40–1.54), and 0.40 (0.15–1.05), respectively. The risk of dementia was 0.68 (0.46–0.99), 0.57 (0.36–0.90), and 0.71 (0.44–1.17) in order of frequency (from low to high).

**Conclusion:**

As an observational study indicated a decreased risk of dementia and PD with long-term TKI use, imatinib and nilotinib may serve as potential therapeutic agents for these conditions, with more evidence from rigorous clinical trials to validate.

## Introduction

The exploration of existing drugs for potential applications to new diseases beyond their original purpose is an interesting issue in drug development, which is commonly known as drug repurposing or repositioning (1, 2). Drug repurposing offers a cost-effective and potentially faster alternative compared to the stages for entirely new drugs, whose success depends on the results of trials: safety profiles, understanding of mechanisms, and sufficient evidence (3).

In this context, nilotinib has emerged as an unexpected candidate for repurposing. Since nilotinib (second generation) was developed as a potent successor to imatinib (first generation), tyrosine kinase inhibitors (TKIs), including imatinib, nilotinib, radotinib, dasatinib, ponatinib, and bosutinib, are now classified within the broader category of targeted cancer therapies. These TKIs are primarily used in the management of chronic myeloid leukemia (CML) caused by the Philadelphia chromosome, which carries the *Bcr-Abl* (*Breakpoint cluster region-Abelson leukemia*) oncogene (4, 5). Recent research has explored the potential application of nilotinib to dementia and Parkinson’s disease (PD) (6, 7). Dementia is characterized by a decline in cognitive function, enough to interfere with daily life (8, 9). Similarly, PD is a neurodegenerative disorder that primarily affects movement and impacts a patient’s daily activities and quality of life because PD is usually accompanied by tremors, stiffness, and difficulty with balance and coordination (10, 11). As there is no definite cure for dementia and PD at present, the search for effective treatments for dementia and PD has become urgent (12, 13). Since both dementia and PD share common pathological mechanisms, there is potential for TKIs to mitigate the risk of both neurodegenerative diseases through shared pathways. As TKIs, not only nilotinib but also radotinib and dasatinib, have been expected as a prospect for the treatment of dementia and PD, several clinical trials have been conducted to explore their therapeutic potential. When the phase 2 trial with 63 participants was conducted in a single center, it suggested the safety of the long-term use of nilotinib in patients with PD (14). Moreover, the randomized controlled phase 2 trial with 300 participants demonstrated that nilotinib is reasonably safe and tolerated in PD patients and has the potential as a new treatment for PD (15). On the other hand, a clinical trial involving 76 participants with moderately advanced PD reported a lack of evidence supporting the efficacy of nilotinib in PD treatment (16). To provide a balanced perspective on the effectiveness or lack of association, real-world evidence from large populations regarding the efficacy of TKIs in dementia and PD remains insufficient. Real-world data, such as insurance claim data, can offer strong generalizability by capturing clinical practices across diverse populations. Their long-term follow-up also enables the assessment of delayed or rare effects that are often missed in clinical trials. Insights may be gained through the observation of pre-existing medical data, rather than relying solely on cost-and time-consuming trial studies.

In this observational cohort study, we investigated evidence suggesting that TKIs, primarily imatinib and nilotinib, may hold promise as potential therapeutic agents for dementia and PD using national insurance data. By assessing the reduced incidence of dementia and PD (prevention) in relation to TKI use and cumulative exposure, we aimed to identify the association between TKIs and outcomes and discuss common pathological mechanisms underlying both prevention and treatment. Ultimately, our study seeks to provide insights into the potential role of TKIs as therapeutic agents for dementia and PD.

## Methods

### Data source

Analyzing real-world information embedded within insurance claim data provides a cost-effective opportunity to conduct research that can reveal evidence to help decision-making and ultimately improve health outcomes. The National Health Insurance Service (NHIS) supports almost all medical practices in Korea (17, 18). The data from the NHIS records offer a comprehensive view of interactions between factors and help to understand hidden patterns. After the review of research ethics and approval, this cohort study used the NHIS data to collect TKI users and analyze the risk of dementia and PD. The Institutional Review Board (IRB) of Seoul National University Hospital (Seoul National University College of Medicine/Hospital Ethics Committee of Medical Research and Center for Human Research Protection) approved this study (E-2403-045-1519). The requirement for informed consent was waived by the IRB as the NHIS database is anonymized according to strict confidentiality guidelines prior to distribution. This retrospective cohort and observation study was conducted by following the Strengthening the Reporting of Observational Studies in Epidemiology (STROBE) guidelines and checklist.

### Study population

Based on the history of drug prescription and diagnosed diseases, the study population whose age was more than 40 years old was recruited in this study. First, 8,679 users who were prescribed Bcr-Abl TKIs within the period (5 years: from January 1, 2013 to December 31, 2017) were recruited. A total of 609 events of death, dementia, or PD were excluded before the index date (January 1, 2018). To ensure the homogeneity of the study population and minimize potential bias, we included only 5,579 cancer patients in the analysis. Prescriptions with other treatment purposes (e.g., for autoimmune diseases) were excluded (N = 2,491) due to heterogeneity in baseline characteristics, mortality rates, and the incidence of dementia and PD, which differ substantially between cancer patients and those treated for other diseases. On the other hand, through the same exclusion criteria, the candidates for the match were recruited among the never-users who had not received Bcr-Abl TKIs by the end of the follow-up (31 December 2022). After the 1:5 propensity score (PS) match was performed (PS for age, sex, income level, Charlson comorbidity index, and year of cancer diagnosis; caliper = 0.1), 5,579 users and 27,895 never-users were observed for the incidence of dementia or PD (Supplementary Figure S1). For the additional analysis with added covariates, 3,922 and 21,441 were used as the users and never-users, respectively, who got national health checkups (2 years: from 1 January 2016 to 31 December 2017).

### Exposure: Bcr-Abl tyrosine kinase inhibitors

Bcr-Abl TKIs included imatinib, nilotinib, radotinib, and dasatinib (19). The starting point of insurance claims for each drug is January 2002 (imatinib), January 2012 (nilotinib), September 2012 (radotinib), May 2008 (dasatinib), and June 2018 (ponatinib; not considered in the study). The accumulated frequency [times] of Bcr-Abl TKIs, which were prescribed between 1 January 2013 and 31 December 2017, was calculated, after the users were classified according to the drug (single and multi-use). Additionally, the risk in single users without multi-class users was evaluated.

### Outcome: dementia and Parkinson’s disease

Based on the verified operational definitions of previous studies, dementia and PD were operatively defined by the International Classification of Diseases Version 10th (ICD-10) code and the history of prescription and medical service. While Parkinson’s disease (PD) was defined as ≥3 outpatient visits with the ICD-10 code “G20” (20, 21), dementia, including Alzheimer’s disease (“F00” and “G30”), vascular dementia (“F01”), and other types, was defined as a new diagnosis with ICD-10 codes (“F00,” “F01,” “F02,” “F03,” and “G30”) in conjunction with prescriptions for anti-dementia medications, including donepezil, rivastigmine, galantamine, or memantine (22). The first day of claiming insurance, which satisfies the above operational definition, was represented as the date of initial diagnosis. All participants were followed up until the end of the study, the date of death, or the date of the new event.

### Statistical analysis

The Cox proportional hazards model was used to evaluate the risk of death by calculating adjusted hazard ratios (aHRs) and 95% confidence intervals (CIs). Since Bcr-Abl TKIs were used for severe diseases such as CML, significantly different mortality in users and never-users was shown. Thus, to evaluate the risk of dementia and PD, adjusted competitive hazard ratios (aCHRs) and 95% CIs from the competitive risk analysis (Fine-Gray Model) were used (23, 24). The covariates for the adjustment included age (continuous; years), sex (categorical), income level (categorical; quartile), Charlson comorbidity index (continuous), type of cancer (categorical), and year of cancer (continuous). Charlson’s comorbidity index was calculated and represented as morbidity. Based on ICD-10 codes, gastrointestinal cancer (GI cancer; “C16-C20”), CML (“C92.1” and “C92.2”), and other cancers of the head–neck, liver, lung, pancreas, breast, prostate, thyroid, and lymphocyte were isolated (25, 26). The year of cancer diagnosis is used as an indicator of the duration of prevalence. Since the incidence of dementia and PD differs across age groups, particularly the elderly as a high-risk population, as well as sex and obesity, stratified analyses were also performed based on age (cutoff: 60 years), sex (men or women), and BMI (cutoff: 25 kg/m^2^ for men and 23 kg/m^2^ for women).

The statistical results are expressed as a number of participants (%) and a mean value ± standard deviation. To compare the differences in the distribution of covariates, statistical significance was defined as an unadjusted *p*-value of <0.05 (two-tailed) when the chi-squared test for categorical variables and analysis of variance (ANOVA) for continuous variables were used. To minimize errors from multiple comparisons, adjusted *p*-values were also calculated using the Benjamini–Hochberg procedure. All data collection and statistical analyses were conducted using SAS 9.4 (SAS Institute Inc., Cary, NC, USA).

## Results

In both 5,579 users and 27,895 never-users (1:5 PS match), the average age was approximately 60.5 years, and the sex ratio (men to women) was similarly identified as 1.4 (Table 1). Although the prevalence of GI cancer was similar (the early 30%), there was a difference in the prevalence of CML: 0.02% in the never-users and 45.9% in the users. The sub-cohort with various records from the national health examination consisted of 21,441 non-users and 3,922 users. Body mass index was similar at approximately 24.1 in both. Table 2 shows that the mean of total usage within 5 years in the users who were prescribed Bcr-Abl TKIs was 859 ± 614 times (days). When the risk of death was evaluated [aHR (95% CIs, unadjusted *p*)], the users had significantly higher mortality compared to non-users [1.41 (1.30–1.54, <0.001)]. Similarly, higher mortality in the users of imatinib [1.44 (1.33–1.57, <0.001)], radotinib [1.63 (1.16–2.30, <0.05)], and dasatinib [1.87 (1.54–2.28, <0.001)] was observed. When the risk of dementia was evaluated, aCHRs (95% CIs, unadjusted and adjusted *p*) for all-typed dementia were 0.65 (0.48–0.88, <0.01 and <0.05), 0.66 (0.48–0.89, <0.01 and <0.05), and 0.46 (0.23–0.93, <0.05 and 0.055) in the users of all-type, imatinib, and nilotinib, respectively. Similarly, aCHRs (95% CIs, unadjusted and adjusted *p*) for Alzheimer’s disease were 0.60 (0.43–0.83, <0.01 and <0.01), 0.60 (0.43–0.84, <0.01 and <0.01), and 0.38 (0.18–0.83, <0.05 and <0.05) in the users of all-type, imatinib, and nilotinib, respectively. Furthermore, lower aCHRs for PD were identified: 0.56 (0.33–0.97, <0.05 and <0.05) and 0.55 (0.32–0.96, <0.05 and <0.05) in the users of all and imatinib, respectively. Supplementary Table S1 showed that the risk of dementia and PD was reduced in users of imatinib and nilotinib when a single user was identified. In the users of nilotinib, the risk of dementia [0.51 (0.19–1.38)] and PD [0.38 (0.04–3.32)] was lower than that in the users of imatinib. When the covariates from the health check-up were more adjusted, aCHRs (95% CIs, unadjusted *p*) in the users decreased compared to that of the never-users: 0.65 (0.44–0.95, <0.05) for all-typed dementia and 0.50 (0.26–0.99, <0.05) for PD (Supplementary Table S2). The risk of dementia and PD was reduced among the older (age≥60 years): aCHRs (95% CIs, *p*) was 0.63 (0.46–0.86, <0.01) for all-typed dementia and 0.37 (0.18–0.75, <0.01) for PD (Supplementary Table S3). The risk of both also decreased among men and women. When all users were divided into three equal groups according to the total usage of Bcr-Abl TKIs, the means of total usage (times ± SD) for 5 years were 201 ± 127, 778 ± 200, and 1,598 ± 297 times in the first (low; *N* = 1,859), second (middle; *N* = 1,860), third (high; *N* = 1,860) of trisection, respectively, (Table 3). The aCHRs for all-typed dementia were 0.68 (0.46–0.99, <0.05), 0.57 (0.36–0.90, <0.05), and 0.71 (0.44–1.17) PD in the low, middle, and high groups, compared to that in the never-users, which *p* for trend in the above four groups was 0.005. Similarly, the aCHRs for PD in the low, middle, and high groups were 0.46 (0.20–1.02), 0.78 (0.40–1.54), and 0.40 (0.15–1.05), respectively. However, there was no statistical difference between the low and high groups’ risk of dementia (*p* = 0.327) and PD (*p* = 0.621).

**Table 1:** Characteristics of the study population.

	Never-user^a^ (Matched)	User of Bcr-Abl TKIs
Study population, N	27,895	5,579
Total usage^b^ [times], mean ± SD (range)		859 ± 614 (1–3,542)
Type of Bcr-Abl TKI [Used], *N* (%)		
Imatinib	0	3,980 (71.3)
Nilotinib	0	1,010 (18.1)
Radotinib	0	300 (5.4)
Dasatinib	0	1,228 (22.0)
Age [years], mean ± SD	60.5 ± 11.1	60.5 ± 11.5
Age [years], *N* (%)		
40–59	13,689 (49.1)	2,760 (49.5)
60–79	12,836 (46.0)	2,519 (45.2)
≥ 80	1,370 (4.9)	300 (5.4)
Sex, *N* (%)		
Men	16,237 (58.2)	3,240 (58.1)
Women	11,658 (41.8)	2,339 (41.9)
Income level, *N* (%)		
Q1	11,027 (39.5)	2,144 (38.4)
Q2	6,519 (23.4)	1,290 (23.1)
Q3	4,675 (16.8)	966 (17.3)
Q4	5,674 (20.3)	1,179 (21.1)
Charlson comorbidity index, *N* (%)		
0–2	9,083 (32.6)	1,692 (30.3)
3–5	12,894 (46.2)	2,655 (47.6)
≥ 6	5,918 (21.2)	1,232 (22.1)
Prevalence of cancer [Yes], *N* (%)		
Gastrointestinal cancer	8,597 (30.8)	1,926 (34.5)
Chronic myelogenous leukemia	6 (0.02)	2,563 (45.9)
Year of the diagnosed date of cancer, *N* (%)		
2010–2011	4,531 (16.2)	1,003 (18.0)
2012–2013	6,147 (22.0)	1,261 (22.6)
2014–2015	7,871 (28.2)	1,538 (27.6)
2016–2017	9,346 (33.5)	1,777 (31.8)
Body mass index^c^ [kg/m^2^], mean ± SD	24.16 (3.26)	24.06 (3.14)
Smoking status^c^, *N* (%)		
Never	12,408 (57.9)	2,335 (59.5)
Former	5,899 (27.5)	1,119 (28.5)
Current	3,134 (14.6)	468 (11.9)
Alcohol consumption^c^ [times/week], *N* (%)		
0–1 (None and Light)	17,285 (80.6)	3,373 (86.0)
2–4 (Moderate)	3,284 (15.3)	443 (11.3)
5–7 (Heavy)	872 (4.1)	106 (2.7)
Physical activity^c^ [times/week], *N* (%)		
0–1 (None and Light)	14,393 (67.1)	2,708 (69.0)
2–4 (Moderate)	5,670 (26.4)	989 (25.2)
5–7 (Heavy)	1,378 (6.4)	225 (5.7)

a Not use any type of Bcr-Abl inhibitors (imatinib, nilotinib, radotinib, dasatinib, and ponatinib) from 1 January 2002 to 31 December 2022.

b Accumulated times of prescribed Bcr-Abl TKIs, between 1 January 2013 and 31 December 2017.

c Participants who take the national health check-up (*N* of never-user = 21,441; N of user = 3,922).

TKIs, tyrosine kinase inhibitors; *N*, number of participants; SD, standard deviation.

**Table 2:** Association of Bcr-Abl TKI use with the incidence of dementia or Parkinson’s disease.

	Never-user	User of Bcr-Abl TKIs				
		All	Imatinib	Nilotinib	Radotinib	Dasatinib
Study population, *N*	27,895	5,579	3,980	1,010	300	1,228
Total usage [times], mean ± SD		859 ± 614	740 ± 613	854 ± 602	477 ± 419	687 ± 545
Death						
Events, *N* (%)	4,309 (15.4)	935 (16.8)	804 (20.2)	98 (9.7)	37 (12.3)	166 (13.5)
Personal year	126,439	25,382	17,729	4,776	1,408	5,621
aHR (95% CIs)	1.00 (Reference)	1.04 (0.97, 1.12)	1.18 (1.09, 1.27)#	0.68 (0.56, 0.83)#	0.90 (0.65, 1.25)	1.00 (0.85, 1.16)
All dementia						
Events, *N* (%)	689 (2.5)	142 (2.6)	118 (3.0)	18 (1.8)	6 (2.0)	23 (1.9)
Personal year	124,970	25,587	17,489	4,733	1,389	5,575
aCHR (95% CIs)	1.00 (Reference)	0.65 (0.48, 0.88)**	0.66 (0.48, 0.89)**	0.46 (0.23, 0.93)*	0.60 (0.23, 1.57)	0.69 (0.36, 1.34)
adjusted *p*-value	Reference	0.017	0.024	0.055	0.460	0.426
Alzheimer’s disease						
Events, *N* (%)	603 (2.2)	119 (2.1)	95 (2.4)	15 (1.5)	5 (1.7)	19 (1.5)
aCHR (95% CIs)	1.00 (Reference)	0.60 (0.43, 0.83)**	0.60 (0.43, 0.84)**	0.38 (0.18, 0.83)*	0.48^a^ (0.16, 1.41)	0.58 (0.28, 1.18)
adjusted *p*-value	Reference	0.008	0.004	0.032	0.338	0.242
Parkinson’s disease						
Events, *N* (%)	253 (0.9)	41 (0.8)	34 (0.8)	1 (0.1)	5 (1.7)	6 (0.5)
Personal year	125,888	25,298	17,664	4772	1,396	5,607
aCHR (95% CIs)	1.00 (Reference)	0.56 (0.33, 0.97)*	0.55 (0.32, 0.96)*	0.20^a^ (0.01, 1.18)	1.79^a^ (0.53, 6.47)	0.52 (0.18, 1.45)
adjusted *p*-value	Reference	0.038	0.031	0.115	0.528	0.388

Cox proportional hazards regression and competing risk (Fine-Gray Model; all-cause death as competitive event) analyses were used to calculate adjusted hazard ratios and 95% confidence intervals after adjustment of the following covariates: age, sex, income level, Charlson comorbidity index, type of cancer, and year of cancer.

a Not enough events (≤ 5).

TKIs, tyrosine kinase inhibitors; N, number of participants; aHR, adjusted hazard ratio; CIs, confidence intervals; aCHR, adjusted competitive hazard ratio.

Unadjusted *p*-value: *p-value<0.05; **p-value<0.01; #*p*-value<0.001 and adjusted p-value through Benjamini–Hochberg adjustments.

**Table 3:** Risk of dementia and Parkinson’s disease, according to the prescription frequency.

	Never-user	User of Bcr-Abl TKIs (All), frequent			*p* for trend
		Low (1st trisection)	Middle (2nd trisection)	High (3rd trisection)	
Study population, *N*	27,895	1,859	1,860	1,860	
Total usage [times], mean ± SD		201 ± 127	778 ± 200	1,598 ± 297	
All dementia					
Events	689 (2.5)	53 (2.8)	45 (2.4)	44 (2.4)	
Personal year	124,970	8,057	8,409	8,621	
aCHR (95% CIs)	1.00 (Reference)	0.68 (0.46, 0.99)*	0.57 (0.36, 0.90)*	0.71 (0.44, 1.17)	0.005
		1.00 (Reference)	0.95 (0.55, 1.65)	1.53 (0.75, 3.10)	0.327
Parkinson’s disease					
Events	253 (0.9)	8 (0.4)	18 (1.0)	16 (0.9)	
Personal year	125,888	8,159	8,455	8,684	
aCHR (95% CIs)	1.00 (Reference)	0.46 (0.20, 1.02)	0.78 (0.40, 1.54)	0.40 (0.15, 1.05)	0.040
		1.00 (Reference)	1.92 (0.68, 5.43)	1.31 (0.24, 7.08)	0.621

Competing risk analysis, Fine-Gray Model, was used to calculate adjusted hazard ratios and 95% confidence intervals after adjustment of the following covariates: age, sex, income level, Charlson comorbidity index, type of cancer, and year of cancer.

TKIs, tyrosine kinase inhibitors; *N*, number of participants; aCHR, adjusted-competitive hazard ratio; CIs, confidence intervals.

Unadjusted *p*-value: *p-value<0.05, **p-value<0.01, and #*p*-value<0.001.

## Discussion

In this nationwide retrospective cohort study, 5-year Bcr-Abl TKI users (*N* of users = 5,579), mainly imatinib and nilotinib, showed a decreased risk of dementia and PD. When only a single user was considered among patients with CML and GI cancer, the risk of dementia was reduced in the users of imatinib and nilotinib, whereas the risk of PD significantly decreased in the users of imatinib.

Since users of TKIs exhibit different mortality rates compared to never-users, the risks of death and incidence of dementia-PD were competitively evaluated among cancer patients, unlike RCTs, which aimed at treating dementia or PD among dementia or PD patients. Nonetheless, our key findings indicate that cumulative prescriptions of nilotinib and imatinib were associated with a reduced incidence in patients with CML and GI cancer, which suggests that the drug may play a preventive role in disease onset and even influence a common biological mechanism underlying both disease development and treatment. Notably, although statistical stability and reliable 95% CIs were limited due to the small number of TKIs users and events, a potential dose–response relationship (from no user to high-frequency users) was observed: *p* = 0.005 for dementia and *p* = 0.040 for PD. The three metabolisms of *α*-synuclein, amyloid β (Aβ), and hyperphosphorylated Tau protein (p-Tau) are suggested as major mechanisms by which TKIs are associated with the lower risk of dementia and PD. α-Synuclein is abundant in presynaptic terminals and plays a role in regulating synaptic function. When α-synuclein is misfolded and aggregates, insoluble aggregation of α-synuclein is associated with several neurodegenerative diseases: α-synucleinopathies (27). Since the aggregation of α-synuclein contributes to the loss of dopaminergic neurons, the prescription of TKIs is linked to the lower risk of neurodegenerative diseases by enhancing the autophagic clearance of α-synuclein (28). For instance, while 885 ng/mL human a-synuclein was measured in total brain lysates from the animal model (A53T mice), the level of a-synuclein (467 ng/mL*, p* < 0.05) significantly decreased in the daily injected group of 10 mg/kg nilotinib for 3 weeks. Next, when amyloid precursor protein and Aβ are excessively produced and accumulated, an abnormal range of Aβ plaques in the brain interferes with communication between neurons and is associated with neuronal damage and death; accumulated Aβ plaques are involved in the development of dementia and PD through neurotoxic and inflammatory responses (29, 30). As TKIs effectively lead to amyloid clearance by ubiquitination of Parkin and activation of Parkin–beclin-1 interaction (31, 32), the prescription of TKIs may be associated with a lower risk of dementia and PD. Moreover, as Tau protein, one of the microtubule-associated proteins, is related to the axonal structure’s stability and dynamics in the neuron (33), dysfunction or the distorted structure of Tau protein is linked to neurological disorders and neurodegeneration: tauopathies. As one of these abnormal changes, p-Tau, which causes Tau protein to detach from microtubules and to form insoluble tangles, leads to cytoskeletal destabilization in neurons and interferes with the transportation of essential molecules and organelles; Tau protein and p-Tau are strongly associated with cognitive decline and the development of dementia and PD (34). Remarkably, as nilotinib and its derivatives could effectively target Tau proteins such as hyperphosphorylation sites (35, 36), TKIs could reduce the risk of dementia and PD as inhibitors of tauopathies. For instance, discoidin domain receptors (DDRs) are one of the receptor tyrosine kinases that are overexpressed in the midbrain of patients with PD (37). Knockdown with short hairpin RNAs or administration of pharmacological DDR inhibitors, including nilotinib, increases dopamine levels and reduces neurotoxic proteins (p-Tau and *α*-synuclein). In a mouse model, partial inhibition or complete deletion of DDR-1 increases autophagy of neurotoxic proteins and reduces inflammation (38). Additionally, a previous study showed that a reduction of p-Tau in nilotinib-treated models enhanced astrocyte activity (39). When nilotinib penetrates the blood–brain barrier, it causes autophagy in neurons to eliminate Tau protein. In the phase 2 trial, the admission of 150 and 300 mg nilotinib showed significantly reduced levels of p-Tau compared to the placebo group: −10.04 pg./mL (*p* < 0.01) in the 150 mg group and −12.05 pg./mL (*p* < 0.01) in the 300 mg group (15). Autophagy clearance of Tau protein, promoted by TKIs, brings the balance of neurotransmitters such as dopamine (40). In addition, during the development of dementia and PD, adverse changes in the immune environment are accomplished, and the activity of neurons is suppressed. TKIs could control the immune system in the central nervous system to ensure the normal activity of neurons (41, 42). In an animal model with TgAPP mice, the formation of Aβ plaques correlated with increased levels of several cytokines (IL-1α/3/6, TNF-α, and IFN-γ) and decreased levels of chemokine (CX3CL1). However, the admission of nilotinib significantly decreased levels of cytokines (IL-6, TNF-α, and IFN-γ) and increased the level of CX3CL1, which maintains neuron–microglia communication.

As TKIs have been widely used in cancer treatment and may be associated with a lower risk of dementia and PD in our study, we should also address the issue of safety. Cardiovascular toxicities and side effects such as hypertension, atrial fibrillation, heart failure, and myocardial infarction have been reported (43), with underlying mechanisms involving endothelial dysfunction, mitochondrial injury, and prothrombotic states. A data-driven cohort study for a drug can offer evidence to evaluate real-world safety profiles (44, 45). Claims databases also cover large and diverse populations over long periods, making it possible to detect rare or long-term adverse events, such as cardiovascular complications, which are often difficult to assess in RCTs due to high costs and limited sample sizes. With analysis for safety, the suggested methodology for drug repurposing with real-world claim data could analyze routine clinical practice with comorbidities, polypharmacy, and medication adherence, as demonstrated in our study, and thus enhance the generalizability of findings. Moreover, longitudinal follow-up allows for time-to-event analyses across patient subgroups. However, unlike RCTs that are carefully designed prospectively prior to execution, retrospective cohort studies using pre-existing data are susceptible to selection bias and unmeasured confounding. For instance, our analysis was restricted to cancer patients who were prescribed or not prescribed TKIs, rather than the general population or elderly individuals. This inherent limitation should be acknowledged when interpreting the findings. Additionally, pre-existing medical data usually lack clinical granularity, such as laboratory values, imaging, or biomarker data, which hampers mechanistic interpretation. Misclassification of diagnoses, drug exposures, or clinical outcomes may also compromise internal validity. Furthermore, evaluating treatment efficacy quantitatively is challenging because treatment responses and symptom improvements are seldom recorded or identifiable. Thus, while the proposed methodology is valuable for identifying safety signals and evaluating real-world evidence for drug repurposing, it is best complemented with other data sources, such as electronic health records, results from *in vitro/vivo* tests, or clinical trial data, to enhance validity and clinical relevance.

For the interpretation of the study, several limitations must be further considered. First, in the NHIS database, the actual prescribed dose (mg) for each patient could not be identified, making it impossible to account for the average prescribed dose (mg) of TKIs. Future studies should aim to incorporate dosage considerations. Due to the insufficient number of their prescribers and events, it was difficult to investigate the association of TKIs, particularly radotinib and dasatinib, with neurodegenerative diseases, as well as to reliably compare outcomes across TKI users of different classes. The results did not reveal significant differences in efficacy between the members of the TKI class, which may rely on binding affinity to other receptors such as c-Kit and platelet-derived growth factor receptors (PDGFRs) (41, 46). Thus, comparing the effectiveness of each class (e.g., imatinib versus nilotinib) was challenging. Furthermore, a detailed analysis of all types of dementia, such as vascular dementia, was not feasible due to the low number of events (fewer than five events), highlighting the need for observation in a larger cohort. Unlike the previous randomized controlled trials of TKIs, which are the gold standard for assessing the potential utility of TKIs, participants (both users and never-users) in our retrospective observational study were not randomly selected and blinded. As TKIs are primarily prescribed for cancer treatment, the user group was composed of 45.9% CML and 34.5% GI cancer, which differs from the matched cancer patients (distribution: 0.02% for CML and 30.8% for GI cancer). Since the potential effects of TKIs have been predominantly evaluated in patients with CML and GI cancer, the observed outcomes may be influenced by predisposing factors, including severity, stage, or type of existing cancer, rather than the direct effects of TKIs. These factors likely stem from the heterogeneity of the study population, including variations in cancer severity and cancer-associated immune or epigenetic environmental influences. Thus, the effects of TKIs have not been extensively studied in the general population or in individuals at high risk of dementia and PD. Based on our selected study population, the factors related to cancer and early death might not be fully controlled, despite the competing risk models, PS match, and adjustment in our study. Especially, our study did not fully consider the patient’s conditions regarding why a certain class of TKI was prescribed across the type, stage, and progression of cancer (severity). Consequently, further studies should handle estimated mortality rates according to drug use and type of cancer and refine selection with additional confounding factors related to patient conditions, such as severity and progression of morbidities.

In conclusion, this national cohort study suggests that Bcr-Abl TKIs, particularly nilotinib and imatinib, may potentially treat dementia and Parkinson’s disease. This conclusion is based on analyses of insurance claim data that included all prescribers of TKIs and tracked them over a long period. Studies focusing on nilotinib and its derivatives for drug repositioning, from cancer treatment to potential breakthroughs in treating degenerative diseases, encourage us to reconsider traditional boundaries in drug development. Although the evidence level is currently low, the potential for new applications of these drugs can be efficiently explored through large-scale cohort data. We hope that research into these unexpected drug applications will continue to advance in the future.

## Data Availability

The data analyzed in this study is subject to the following licenses/restrictions: the raw data used in this study are only accessible to the qualified researchers in permitted security facilities for a certain period, since the used database was based on the national clinical and medical records. Thus, the raw data cannot be shared openly. Unless it deviates from the national and organizational regulations, access to the code used in this study is available, only for non-commercial and academic purposes. Requests to access these datasets should be directed to JS, jih2616@naver.com.

## Supplementary material

Supplementary FIGURE S1 Study flow

Supplementary TABLE S1 Sensitive analysis (single use): the risk of dementia or Parkinson’s disease among the users of TKIs.

Supplementary TABLE S2 Stratified analysis (sub-cohort): the risk of dementia or Parkinson’s disease in the never user and User.

Supplementary TABLE S3 Stratified analysis: a subgroup of age, sex, and body mass index.
