# Supplementary material for: Nilotinib and imatinib: potential candidates for treatment of dementia and Parkinson’s disease through national health insurance data
Source: Front Neurol. 2025 Aug 26;16:1628876. doi: 10.3389/fneur.2025.1628876 (PMC12420838; doi:10.3389/fneur.2025.1628876)
Supplement: Supplementary file 1 [file Supplementary_file_1.docx]

**
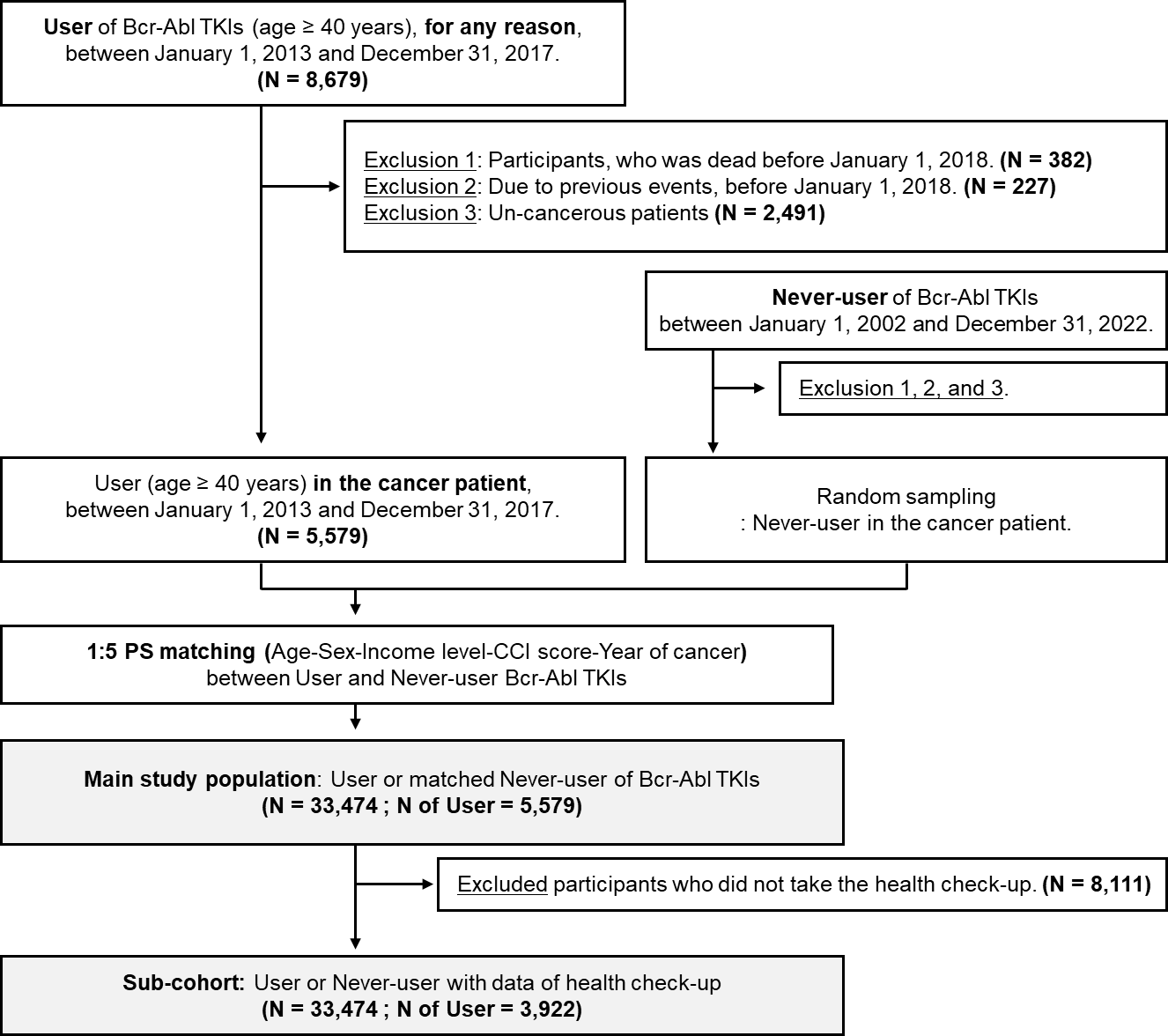
**

**Supplementary information 1. Study flow**

**Supplementary information 2. Sensitive analysis (single use): the risk of dementia or Parkinson’s disease among the users of TKIs.**

|  | **Never-user** | **Single User of Bcr-Abl TKIs** | | | |
| --- | --- | --- | --- | --- | --- |
|  |  | **Imatinib** | **Nilotinib** | **Radotinib** | **Dasatinib** |
| **Study population, N** | 27,895 | 3,464 | 582 | 148 | 628 |
| **Total usage [times], mean ± SD** |  | 798 ± 618 | 1,055 ± 576 | 451 ± 348 | 842 ± 566 |
| **The risk of all dementia** |  |  |  |  |  |
| aCHR (95% CIs) | 1.00 (Reference) | 0.66 (0.48, 0.89)** | 0.37 (0.14, 0.97)* | 0.95^N^ (0.28, 3.17) | 0.66 (0.28, 1.54) |
| adjusted *p*-value | Reference | 0.024 | 0.048 | 0.928 | 0.536 |
| aCHR (95% CIs) |  | 1.00 (Reference) | 0.51 (0.19, 1.38) | 1.12^N^ (0.31, 4.02) | 0.79 (0.32, 1.98) |
| adjusted *p*-value |  | Reference | 0.442 | 0.861 | 0.827 |
| **The risk of Parkinson’s disease** |  |  |  |  |  |
| aCHR (95% CIs) | 1.00 (Reference) | 0.56 (0.32, 0.98)* | 0.20^N^ (0.02,1.56) | 3.32^N^ (0.81, 13.6) | 1.02^N^ (0.33, 3.12) |
| adjusted *p*-value | Reference | 0.231 | 0.348 | 0.354 | 0.979 |
| aCHR (95% CIs) |  | 1.00 (Reference) | 0.38^N^ (0.04, 3.32) | 4.72^N^ (0.88, 25.3) | 1.53^N^ (0.39, 6.01) |
| adjusted *p*-value |  | Reference | 1.000 | 0.421 | 0.935 |

Competing risk analysis, Fine-Gray Model (all-cause death as competitive event), was used to calculate adjusted hazard ratios and 95% confidence intervals after adjustment of the following covariates: Age, Sex, Income level, Charlson comorbidity index, Type of cancer, and Year of cancer.

^N^Not enough events (≤ 5).

Abbreviation: TKIs, tyrosine kinase inhibitors; N, number of participants; BMI, body mass index; aCHR, adjusted-competitive hazard ratio; CIs confidence intervals.

Unadjusted *p*-value: **p*-value<0.05, ***p*-value<0.01, and #*p*-value<0.001 and adjusted *p*-value through Benjamini-Hochberg adjustments.

**Supplementary information 3. Stratified analysis (sub-cohort): the risk of dementia or Parkinson’s disease in the Never‑user and User.**

|  | **Sub-cohort: those who take the national health examination** | | | | | |
| --- | --- | --- | --- | --- | --- | --- |
|  | **Never-user** | **User of Bcr-Abl TKIs** | | | | |
|  |  | **All** | **Imatinib** | **Nilotinib** | **Radotinib** | **Dasatinib** |
| **Study population, N** | 21,441 | 3,922 | 2,723 | 714 | 200 | 767 |
| **Total usage [times], mean ± SD** |  | 833 ± 608 | 718 ± 604 | 850 ± 594 | 444 ± 389 | 695 ± 543 |
| **All dementia** |  |  |  |  |  |  |
| Events, N (%) | 481 (2.2) | 85 (2.2) | 69 (2.5) | 8 (1.1) | 2 (0.9) | 17 (2.0) |
| Personal year | 97,839 | 18,002 | 12,254 | 3,391 | 1,045 | 3,984 |
| aCHR (95% CIs) |  |  |  |  |  |  |
| Model 1 | 1.00 (Reference) | 0.64 (0.44, 0.94)* | 0.65 (0.44, 0.94)* | 0.37 (0.15, 0.96)* | 0.32^N^ (0.07, 1.42) | 1.12 (0.49, 2.56) |
| Model 2 | 1.00 (Reference) | 0.65 (0.44, 0.95)* | 0.65 (0.44, 0.95)* | 0.38 (0.15, 0.96)* | 0.32^N^ (0.07, 1.43) | 1.13 (0.49, 2.60) |
| **Parkinson’s disease** |  |  |  |  |  |  |
| Events, N (%) | 189 (0.9) | 26 (0.7) | 19 (0.7) | 1 (0.1) | 4 (1.8) | 4 (0.5) |
| Personal year | 98,460 | 18,124 | 12,353 | 3,409 | 1,042 | 4,010 |
| aCHR (95% CIs) |  |  |  |  |  |  |
| Model 1 | 1.00 (Reference) | 0.51 (0.26, 0.98)* | 0.48 (0.24, 0.94)* | 0.12^N^ (0.01, 0.96)* | 2.52^N^ (0.60, 10.6) | 0.54^N^ (0.16, 1.81) |
| Model 2 | 1.00 (Reference) | 0.50 (0.26, 0.99)* | 0.48 (0.24, 0.94)* | 0.12^N^ (0.01, 0.99)* | 2.63^N^ (0.62, 11.2) | 0.54^N^ (0.16, 1.82) |

Cox proportional hazards regression and competing risk (Fine-Gray Model) analysis were used to calculate adjusted hazard ratios and 95% confidence intervals after adjustment of the following covariates:

Model 1: Age, Sex, Income level, Charlson comorbidity index, Type of cancer, and Year of cancer.

Model 2: Model 1 + Body mass index, Smoking status, Alcohol consumption, and Physical activity.

^N^Not enough events (≤ 5).

Abbreviation: TKIs, tyrosine kinase inhibitors; N, number of participants; aHR, adjusted hazard ratio; CIs, confidence intervals; aCHR, adjusted-competitive hazard ratio.

Unadjusted *p*-value: **p*-value<0.05, ***p*-value<0.01, and #*p*-value<0.001.

**Supplementary information 4. Stratified analysis: a subgroup of age, sex, and body mass index.**

|  |  | **Never-user** | **User (All)** |  | **Never-user** | **User (All)** |
| --- | --- | --- | --- | --- | --- | --- |
| **Age** |  |  |  |  |  |  |
| Study population, N | **1. 40‑59 years** | 13,689 | 2,769 | **2. ≥ 60 years** | 14,206 | 2,819 |
| Total usage [times], mean ± SD |  |  | 878 ± 617 |  |  | 841 ± 611 |
| The risk of, aCHR (95% CIs) |  |  |  |  |  |  |
| All dementia |  | 1.00 (Reference) | 1.11 (0.33, 3.71) |  | 1.00 (Reference) | 0.63 (0.46, 0.86)** |
| Parkinson’s disease |  | 1.00 (Reference) | 1.82 (0.80, 4.11) |  | 1.00 (Reference) | 0.37 (0.18, 0.75)** |
| **Sex** |  |  |  |  |  |  |
| Study population, N | **1. Men** | 16,237 | 3,240 | **2. Women** | 11,658 | 2,339 |
| Total usage [times], mean ± SD |  |  | 886 ± 609 |  |  | 823 ± 618 |
| The risk of, aCHR (95% CIs) |  |  |  |  |  |  |
| All dementia |  | 1.00 (Reference) | 0.79 (0.52, 1.21) |  | 1.00 (Reference) | 0.55 (0.36, 0.85)** |
| Parkinson’s disease |  | 1.00 (Reference) | 0.55 (0.26, 1.18) |  | 1.00 (Reference) | 0.55 (0.25, 1.19) |
| **Body mass index** |  |  |  |  |  |  |
| Study population, N | **1. Low**  **Men < 25 kg/m^2^**  **Women < 23 kg/m^2^** | 11,463 | 2,159 | **2. High**  **Men ≥ 25 kg/m^2^**  **Women ≥ 23 kg/m^2^** | 9,978 | 1,763 |
| Total usage [times], mean ± SD |  |  | 830 ± 604 |  |  | 837 ± 612 |
| The risk of, aCHR (95% CIs) |  |  |  |  |  |  |
| All dementia |  | 1.00 (Reference) | 0.72 (0.43, 1.21) |  | 1.00 (Reference) | 0.54 (0.31, 0.95)* |
| Parkinson’s disease |  | 1.00 (Reference) | 0.60 (0.26, 1.38) |  | 1.00 (Reference) | 0.40 ( 0.13, 1.18) |

Competing risk analysis, Fine-Gray Model, was used to calculate adjusted hazard ratios and 95% confidence intervals after adjustment of the following covariates: Age, Sex, Income level, Charlson comorbidity index, Type of cancer, and Year of cancer.

Abbreviation: N, number of participants; aCHR, adjusted-competitive hazard ratio; CIs, confidence intervals.

Unadjusted *p*-value: **p*-value<0.05, ***p*-value<0.01, and #*p*-value<0.001.
